# Impact of diabetes mellitus on the early-phase arterial healing after drug-eluting stent implantation

**DOI:** 10.1186/s12933-020-01173-7

**Published:** 2020-12-02

**Authors:** Takayuki Ishihara, Yohei Sotomi, Takuya Tsujimura, Osamu Iida, Tomoaki Kobayashi, Yuma Hamanaka, Takashi Omatsu, Yasushi Sakata, Yoshiharu Higuchi, Toshiaki Mano

**Affiliations:** 1 Kansai Rosai Hospital Cardiovascular Center, 3-1-69 Inabaso, Amagasaki, 660-8511 Hyogo Japan; 2 Department of Cardiovascular Medicine, Osaka University Graduate School of Medicine, Suita, Japan; 3 Department of Cardiology, Osaka Police Hospital, Osaka, Japan

**Keywords:** Coronary angioscopy, Diabetes mellitus, Drug-eluting stent

## Abstract

**Background:**

Early arterial healing after drug-eluting stent (DES) implantation may enable short dual-antiplatelet therapy (DAPT) strategy. The impact of diabetes mellitus (DM) on this healing has not been elucidated. We used coronary angioscopy (CAS) to compare intravascular status of DM and non-DM patients in the early phase after DES implantation.

**Methods:**

This study was a multicenter retrospective observational study. We analyzed CAS findings of 337 lesions from 270 patients evaluated 3–5 months after DES implantation. We divided the lesion into two groups: DM (n = 149) and non-DM (n = 188). We assessed neointimal coverage (NIC) grades (dominant, maximum and minimum), thrombus adhesion and maximum yellow color grade. NIC was graded as follows: grade 0, stent struts were not covered; grade 1, stent struts were covered by thin layer; grade 2, stent struts were buried under neointima. Yellow color was graded as grade 0, white; grade 1, light yellow; grade 2, yellow; grade 3, intensive yellow.

**Results:**

Minimum NIC grade was significantly lower in DM than in non-DM groups (p = 0.002), whereas dominant and maximum NIC grades were similar between them (p = 0.59 and p = 0.94, respectively), as were thrombus adhesion (44.3% vs. 38.8%, p = 0.32) and maximum yellow color grade (p = 0.78). A multivariate analysis demonstrated that DM was an independent predictor of minimum NIC of grade 0 (odds ratio: 2.14, 95% confidence interval: 1.19–3.86, p = 0.011).

**Conclusions:**

DM patients showed more uncovered struts than non-DM patients 3–5 months after DES implantation, suggesting that the recent ultra-short DAPT strategy might not be easily applied to DM patients.

## Background

Diabetes mellitus (DM) is one of the most considerable risk factors for major adverse cardiac and cerebrovascular events [1]. Patients with coronary artery disease complicated with DM often have complex lesions, and the incidences of peri-operative and long-term adverse events are relatively high [2]. Features of DM, particularly hyperglycemia, free fatty acids, and insulin resistance, provoke molecular mechanisms that alter the function and structure of blood vessels, including increased oxidative stress, disturbances of intracellular signal transduction, and activation of the receptor for advanced glycation end products [3]. Consequently, there is a decreased availability of nitric oxide, an increased production of endothelin, the activation of transcription factors such as NF-κB and AP-1, and an increased production of pro-thrombotic factors such as tissue factor and plasminogen activator inhibitor-1 [3]. These abnormalities contribute to the cellular events such as vasoconstriction, inflammation, and thrombosis that cause atherosclerosis and subsequently increase the risk of the adverse cardiovascular events in individuals with DM [3].

Percutaneous coronary intervention (PCI) using a drug-eluting stent (DES) has been widely applied for patients with coronary artery disease, and dual-antiplatelet therapy (DAPT) is performed in order to prevent stent thrombosis. Although the duration of DAPT after stent implantation has become shorter, it is necessary to consider the risks of both bleeding risk and thrombosis based on the patient’s background [4, 5]. The assessment of arterial healing after DES implantation is also important as it contributes to the decision whether to switch DAPT to single-antiplatelet therapy (SAPT). Early arterial healing may enable a short-DAPT strategy.

Among the intravascular imaging methods that can be used to evaluate arterial healing *in vivo*, coronary angioscopy (CAS) is the only method that enables the observation of the patient’s intravascular status with direct and full-color vision [6–15]. The impact of DM on the safety of switching from DAPT to SAPT in the early phase has not been elucidated, and there has been no investigation of the impact of DM on the patients’ early-phase arterial healing. We used CAS in the present study to determine the effects of DM on early-phase arterial healing after the implantation of a DES.

## Methods

### Patients

This was a multicenter, retrospective, observational study. From the database of CAS evaluations at each hospital, we extracted 337 lesions from 270 patients for which a CAS evaluation was performed 3–5 months after DES implantation. All DESs were implanted in *de novo* lesions in native coronary arteries. Basically, this study included an all-comer population. However, although angioscopic evaluation at follow-up angiography as well as staged PCI for other lesions was recommended for all patients, this was not performed when informed consent could not be obtained, or when a specialist for angioscopic evaluation was not available. The elective patients received ticlopidine (200 mg/day), clopidogrel (75 mg/day), or prasugrel (3.75 mg/day) in addition to aspirin (100 mg/day) at least 1 week before PCI. For the emergent patients, the antiplatelet drugs (aspirin at 200 mg and clopidogrel 300 mg or prasugrel 20 mg) were loaded before PCI. The Medical Ethics Committees of Osaka Police Hospital and Kansai Rosai Hospital approved this study, and all patients provided written informed consent to participate.

### Angioscopic follow-up

CAS was performed after the administrations of unfractionated heparin (5,000 IU) into the radial or femoral artery via the inserted sheath, and isosorbide dinitrate into the coronary artery. At Osaka Police Hospital, a non-occlusion angioscopy device named VISIBLE (FiberTech Co., Ltd., Tokyo, Japan), was used. Angioscopic observation of the stented lesions was carried out while blood was cleared away from the viewing area by the injection of 3% dextran-408 [6]. At Kansai Rosai Hospital, CAS was subsequently performed as previously described using a Fullview NEO angioscopic catheter (FiberTech) during the period from January 2010 to September 2016 [7, 8]. Briefly, an optical fiber was placed at the distal segment of the coronary artery and manually pulled back from the distal edge of the stent to the proximal edge under careful angioscopic and angiographic guidance. Since October 2016, we have been using a smart-i angioscopic catheter (Surgetech Corp., Tokyo, Japan) because the Fullview NEO was discontinued. Using guide extension catheters such as GuideLiner (Japan Lifeline, Tokyo, Japan), Guidezilla (Boston Scientific, Natrick, MA, USA) and Guideplus (NIPRO, Osaka, Japan), we blocked blood flow by flushing with low molecular weight dextran. Both angioscopic images consisted of 3,000 pixels in full color and were digitally stored for off-line analysis [9]. A smart-i 6K angioscopic catheter (Surgetech), which can project images with 6,000 pixels, has been available since October 2018, and this catheter was used in some cases after that time.

### Angioscopic analysis

Angioscopic images were analyzed for each lesion to determine (1) the dominant, maximum, and minimum degree of neointimal coverage (NIC) over the stent; (2) the yellow color grade of the stented segment; and (3) the presence of an intra-stent thrombus. Neointimal coverage over the stent was classified into three grades as previously described: grade 0, the stent struts were not covered by neointima and were fully visible, similar to their status immediately after implantation; grade 1, the stent struts were visible on the surface but were covered by a thin layer; grade 2, the stent struts were not visible under neointima or the stent struts were visible through the neointima but were below the level of the neointimal surface [10]. The yellow color was graded as follows: grade 0, white; grade 1, light yellow; grade 2, yellow; grade 3, intensive yellow [11]. Thrombus was defined based on the criteria adopted by the European Working Group on Coronary Angioscopy [12]. As mentioned in reports from each institution, the reproducibility was as follows: (1) Osaka Police Hospital: the inter-observer and intra-observer reproducibility (percent agreement) values for the interpretation of angioscopic images at this institution were 95% and 95% for stent coverage, 85% and 95% for plaque color, and 90% and 100% for thrombus, respectively [13, 14]; (2) Kansai Rosai Hospital: the estimated inter- and intra-observer κ coefficients were 84% and 95%, respectively for the dominant degree of NIC over the stent, 82% and 86% for the yellow color grade of the stented segment and 93% and 100% for the presence of intra-stent thrombus [9].

### Quantitative coronary angiography (QCA)

Coronary angiography was performed in at least ten projections. The view showing the most severe stenosis was selected for QCA, which was subsequently performed using a computerized angiographic analysis system (CAAS Workstation 5.11, Pie Medical Imaging, Maastricht, The Netherlands) at the same angle of projection prior to and immediately after PCI [15].

### Outcome measures

DM was defined as the use of an oral agent or insulin treatment for DM or an HbA_1C_ value ≥ 6.5%. We compared the CAS outcomes between the DM and non-DM patients, and we performed a multivariate analysis to identify the outcome(s) that showed a significant between-group difference. In the DM group, the relationship between the outcomes and the baseline characteristics was examined.

### Statistical analyses

All results are expressed as the mean ± SD unless otherwise stated. Continuous variables with and without homogeneity of variance were analyzed by Student’s t-test and the Welch t-test, respectively. Categorical variables were analyzed with Fisher’s exact test for 2 × 2 comparisons. For more than 2 × 2 comparisons, nominal variables and ordinal variables were analyzed with the Chi-squared test and the Mann-Whitney test, respectively. The multivariate analysis was performed with a logistic regression analysis. Variables in the univariate analysis with p-values < 0.1 were selected for the multivariate analysis. Statistical significance was defined as p < 0.05. All calculations were performed using the IBM SPSS Statistics package ver. 24 (IBM Corp., Armonk, NY, USA).

## Results

### Baseline characteristics

The DM group was comprised of 149 lesions from 118 patients. The characteristics of the patients in the DM and non-DM groups are summarized in Table 1. There was no significant difference in characteristics between the groups. The uses of medication at the time of PCI and at CAS evaluations are shown in Table 2. Although the usage of a statin tended to be lower in the DM group compared to the non-DM groups, there were no significant between-group differences in medication use except for the hypoglycemic agents. More than 90% of the total series of patients received DAPT with aspirin and P2Y12 inhibitor at the time of CAS evaluation. Table 3 provides the groups’ laboratory data at the time points of PCI and CAS evaluation. At the time point of PCI, although the HbA_1c_, fasting blood sugar values, and the triglyceride level were significantly higher in the DM group, the high-density lipoprotein value was significantly higher in the non-DM group. Total cholesterol was significantly higher in the non-DM group at the time of the CAS evaluation. Table 4 summarizes the lesion and procedural characteristics. Among the lesion characteristics, the follow-up duration was significantly shorter and the rate of acute coronary syndrome was significantly lower in the DM group compared to the non-DM group. Regarding the procedural characteristics, the minimum stent diameter was significantly smaller in the DM group than in non-DM group. Various types of DES were used in this patient series, and the type of DES was not significantly different between the DM and non-DM groups.

**Table 1:** Patient characteristics

	DM (n = 118)	Non-DM (n = 152)	p value
Male, n (%)	101 (86)	127 (84)	0.74
Age, years	67 ± 11	69 ± 10	0.11
Hypertension^a^, n (%)	105 (89)	125 (82)	0.17
Dyslipidemia^b^, n (%)	92 (78)	125 (82)	0.44
Smoking status, n (%)			0.11
Non-smoker	52 (44)	81 (53)	
Current smoker	30 (25)	41 (27)	
Past smoker	36 (31)	30 (20)	
SYNTAX score	11.2 ± 8.8	11.1 ± 7.0	0.87

Data are presented as mean ± SD or number (%)

*DM* diabetes mellitus

^a^Receiving antihypertensive medication, systolic blood pressure ≥ 140 mmHg, or diastolic blood pressure ≥ 90 mmHg.

^b^Treatment with medication, total cholesterol ≥ 220 mg/dL, low-density lipoprotein cholesterol ≥ 140 mg/dL, high-density lipoprotein cholesterol ≤ 40 mg/dL, or triglycerides ≥ 150 mg/dL

**Table 2:** Medication use

	DM (n = 149)	Non-DM (n = 188)	p value
At the time of PCI			
Aspirin, n (%)	139 (93)	173 (92)	0.41
Clopidogrel, n (%)	73 (49)	112 (60)	0.061
Prasugrel, n (%)	52 (35)	53 (28)	0.20
Ticlopidine, n (%)	7 (5)	8 (4)	1.0
Statin, n (%)	80 (54)	92 (49)	0.22
Insulin, n (%)	24 (16)	0 (0)	N/A
Biguanide, n (%)	38 (26)	0 (0)	N/A
DPP-4 inhibitor, n (%)	59 (40)	0 (0)	N/A
SGLT-2 inhibitor, n (%)	4 (3)	0 (0)	N/A
Thiazolidine, n (%)	18 (12)	0 (0)	N/A
Sulfonylurea, n (%)	39 (26)	0 (0)	N/A
Glinide, n (%)	6 (4)	0 (0)	N/A
α-glucosidase inhibitor, n (%)	26 (17)	0 (0)	N/A
At the time of CAS evaluation			
Aspirin, n (%)	149 (100)	186 (99)	0.31
Clopidogrel, n (%)	79 (53)	104 (55)	0.74
Prasugrel, n (%)	63 (42)	64 (34)	0.14
Ticlopidine, n (%)	6 (4)	8 (4)	1.0
Statin, n (%)	102 (69)	142 (76)	0.094
Insulin, n (%)	25 (17)	0 (0)	N/A
Biguanide, n (%)	43 (29)	0 (0)	N/A
DPP-4 inhibitor, n (%)	62 (42)	0 (0)	N/A
SGLT-2 inhibitor, n (%)	5 (3)	0 (0)	N/A
Thiazolidine, n (%)	18 (12)	0 (0)	N/A
Sulfonylurea, n (%)	40 (27)	0 (0)	N/A
Glinide, n (%)	10 (7)	0 (0)	N/A
α-glucosidase inhibitor, n (%)	31 (21)	0 (0)	N/A

Data are presented as number (%)

* CAS * coronary angioscopy,* DM*  diabetes mellitus,* DPP-4* dipeptidyl peptidase-4,* N/A * not available, * SGLT-2 * sodium glucose cotransporter-2

**Table 3:** Laboratory Data

	DM (n = 149)	Non-DM (n = 188)	p value
At the time of PCI			
HbA_1c_, %	7.1 ± 1.2	5.8 ± 0.5	< 0.001
FBS, mg/dL	156 ± 60	110 ± 30	< 0.001
Total cholesterol, mg/dL	176 ± 39	180 ± 38	0.25
Triglyceride, mg/dL	169 ± 109	133 ± 100	0.002
HDL-cholesterol, mg/dL	44 ± 13	50 ± 14	< 0.001
LDL-cholesterol, mg/dL	103 ± 32	105 ± 34	0.58
CRP, mg/dL	0.42 ± 1.04	0.71 ± 2.53	0.17
Hemoglobin, g/dL	13.5 ± 2.1	13.7 ± 1.7	0.45
Creatinine, mg/dL	1.2 ± 1.9	1.1 ± 1.1	0.31
eGFR, mL/min/m^2^	65 ± 21	64 ± 18	0.39
Uric acid, mg/dL	5.6 ± 1.5	5.9 ± 1.5	0.052
At the time of CAS evaluation			
HbA1c, %	6.8 ± 0.8	5.7 ± 0.5	< 0.001
FBS, mg/dL	146 ± 55	107 ± 21	< 0.001
Total cholesterol, mg/dL	162 ± 35	170 ± 31	0.036
Triglyceride, mg/dL	151 ± 112	141 ± 100	0.36
HDL-cholesterol, mg/dL	47 ± 18	50 ± 13	0.057
LDL-cholesterol, mg/dL	90 ± 26	92 ± 25	0.46
CRP, mg/dL	0.24 ± 0.52	0.32 ± 1.25	0.49
Hemoglobin, g/dL	13.2 ± 1.9	13.6 ± 1.6	0.036
Creatinine, mg/dL	1.5 ± 3.8	1.1 ± 0.9	0.19
eGFR, mL/min/m2	64 ± 18	60 ± 17	0.14
Uric acid, mg/dL	5.6 ± 1.5	5.9 ± 1.5	0.073

Data are presented as mean ± SD

* CAS * coronary angiography,* CRP*   C-reactive protein,* DM* diabetes mellitus,* eGFR*  estimated glomerular filtration rate,* FBS* fasting blood sugar, HbA_1c_  Hemoglobin A_1c_,* HDL *high-density lipoprotein,* LDL*  low-density lipoprotein

**Table 4:** Lesion and procedural characteristics

	DM (n = 149)	Non-DM (n = 188)	p value
Follow-up duration, days	115 ± 29	121 ± 25	0.031
Evidence of ischemia, n (%)			0.006
Exercise tests	6 (4)	12 (6)	
Scintigraphy	25 (17)	17 (9)	
FFR/iFR	12 (8)	4 (2)	
None	106 (71)	155 (82)	
Acute coronary syndrome, n (%)	45 (30)	78 (42)	0.040
Target vessel, n (%)			0.18
Left anterior descend artery	44 (30)	66 (35)	
Left circumflex artery	64 (43)	85 (45)	
Right coronary artery	35 (23)	27 (15)	
Left main trunk	6 (4)	10 (5)	
Heavy calcification, n (%)	17 (11)	19 (10)	0.42
Bifurcation, n (%)	53 (36)	73 (39)	0.31
Chronic total occlusion, n (%)	4 (3)	13 (7)	0.063
ACC/AHA classification, n (%)			
Type A/ B1/ B2/ C	15 (10)/ 26 (17) / 24 (16)/ 84 (56)	20 (11)/ 34 (18) / 41 (22)/ 93 (50)	0.53
Pre-dilatation balloon			
Maximum diameter, mm	2.63 ± 0.53	2.66 ± 0.52	0.73
Maximum pressure, atm	13 ± 4	13 ± 3	0.78
Maximum stent diameter, mm	3.04 ± 0.45	3.12 ± 0.40	0.069
Minimum stent diameter, mm	2.92 ± 0.43	3.02 ± 0.43	0.030
Total stent length, mm	31 ± 16	31 ± 20	0.86
Stent implantation pressure, atm	12 ± 3	12 ± 3	0.85
Post-dilatation balloon			
Maximum diameter, mm	3.20 ± 0.56	3.28 ± 0.65	0.28
Maximum pressure, atm	17 ± 4	16 ± 4	0.31
Number of stents	1.2 ± 0.4	1.2 ± 0.4	0.91
Usage of intravascular imaging device, n (%)			0.19
Intravascular ultrasound	118 (79)	162 (86)	
Optical coherence tomography	28 (19)	22 (12)	
None	3 (1)	4 (1)	
Type of DES			0.21
Cypher	1 (1)	2 (1)	
Taxus	0 (0)	1 (1)	
Endeavor	28 (19)	50 (27)	
Xience	30 (20)	32 (17)	
Promus	0 (0)	4 (2)	
Resolute	35 (23)	38 (20)	
Synergy	26 (17)	24 (13)	
Ultimaster	21 (14)	23 (12)	
Orsiro	0 (0)	3 (2)	
BioFreedom	8 (5)	11 (6)	
QCA data			
Pre-PCI			
Minimum lumen diameter, mm	0.95 ± 0.57	0.86 ± 0.57	0.14
Reference diameter, mm	2.65 ± 0.69	2.78 ± 0.80	0.11
Diameter stenosis, %	64 ± 20	70 ± 19	0.020
Lesion length, mm	15 ± 9	17 ± 12	0.13
Post-PCI			
Minimum lumen diameter, mm	2.39 ± 0.52	2.52 ± 0.51	0.028
Reference diameter, mm	2.85 ± 0.55	3.03 ± 0.59	0.005
Diameter stenosis, %	16 ± 8	17 ± 8	0.50

Data are presented as mean ± SD or number (%)

*DES*  drug-eluting stent,* DM* diabetes mellitus,* FFR * fractional flow reserve,* iFR*  instantaneous wave-free ratio,* PCI * percutaneous coronary intervention,* QCA * quantitative coronary angiography

## Angioscopic findings

**Fig. 1:**
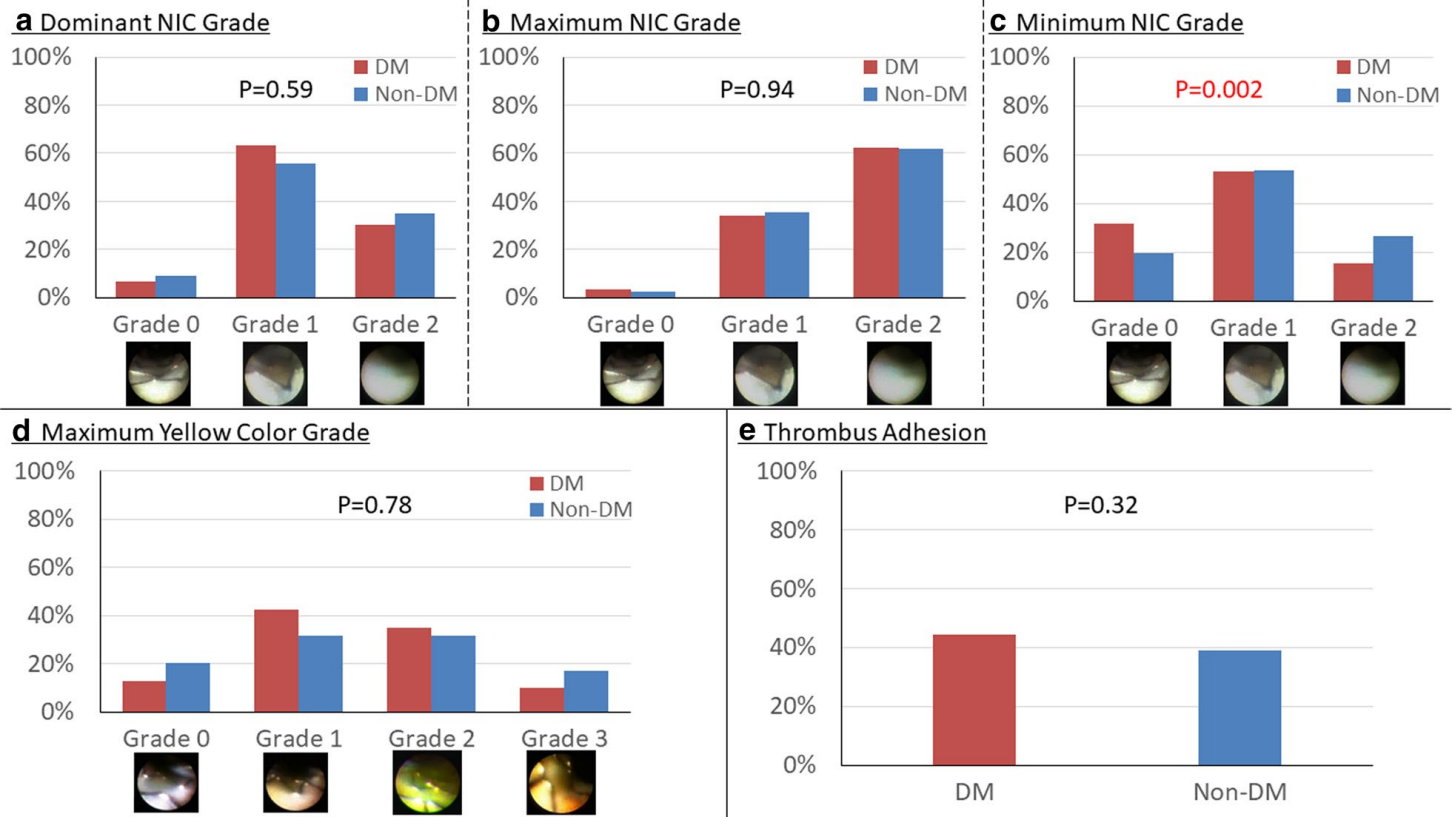
Coronary angioscopic findings 3–5 months after the Implantation of a drug-eluting stent **a** Dominant NIC grade. The dominant NIC grade was similar between the DM and non-DM groups (p = 0.59). **b**Maximum NIC grade. The maximum NIC grade was similar between the DM and non-DM groups (p = 0.94). **c** Minimum NIC grade. The minimum NIC grade was significantly lower in the DM group than in the non-DM group (p = 0.002). In the DM group, 47 lesions (32%) demonstrated the minimum NIC of grade 0. **d** Maximum yellow color grade. The maximum yellow color grade was similarly distributed between the DM and non-DM groups (p = 0.78). **e**Thrombus Adhesion. The rate of thrombus adhesion was similar between the DM and non-DM groups (44% vs. 39%, p = 0.32). *DM* diabetes mellitus,* NIC* neointimal coverage

The details of the patients’ angioscopic findings are illustrated in Fig. 1. The minimum NIC grade was significantly lower in the DM group than in the non-DM group, but the dominant and maximum NIC grades were similar between the groups. In the DM group, 47 lesions (32%) demonstrated the minimum NIC of grade 0. The maximum yellow color grade was similarly distributed between the DM and non-DM groups. Thrombus adhesion was similar between the DM and non-DM groups (44% vs. 39%, p = 0.32). The multivariate analysis conducted to detect the independent predictors of the minimum NIC of grade 0 revealed that while DM (odds ratio [OR]: 1.88, 95% confidence interval [CI] 1.14–3.10], p = 0.016), the maximum diameter of the post-dilatation balloon (OR: 0.51, 95% CI 0.32–0.83, p = 0.006), and the maximum inflation pressure of the post-dilatation balloon (OR: 0.93, 95% CI 0.87–0.99, p = 0.048) were significant predictors in the univariate analysis, only DM (OR: 2.14, 95%CI 1.19–3.86, p = 0.011) and the maximum diameter of the post-dilatation balloon (OR: 0.51, 95% CI 0.31–0.86, p = 0.011) were independent predictors even after the multivariate analysis (Table 5). We assessed the independent predictors for the minimum NIC of grade 0 in the DM group (Table 6). The univariate analysis showed that the usage of sulfonylurea at the time of the CAS evaluation was significantly associated with the minimum NIC of grade 0. Even after the adjustment for the usage of a statin (OR: 2.44, 95% CI 1.01–5.87, p = 0.047) at the time of the CAS evaluation, the following were observed to be independent predictors of the minimum NIC of grade 0: the stent implantation pressure (OR: 0.87, 95% CI 0.76–0.99, p = 0.040) and low-density lipoprotein cholesterol at the time of PCI (OR: 0.984, 95% CI 0.972–0.997, p = 0.018), and the use of sulfonylurea (OR: 3.87, 95% CI 1.66–9.01, p = 0.002).

**Table 5:** Logistic regression analysis for minimum NIC of grade 0 (Uncoverage)

	Univariate			Multivariate		
	OR	95% CI	p value	OR	95% CI	p value
Male	1.39	0.64–2.84	0.48			
Age (1 year increase)	1.00	0.98–1.03	0.98			
BMI (1 kg/m^2^ increase)	1.03	0.96–1.09	0.44			
Hypertension	0.83	0.42–1.63	0.60			
Dyslipidemia	1.39	0.73–2.65	0.35			
DM	1.88	1.14–3.10	0.016	2.14	1.19–3.86	0.011
Aspirin*	0.33	0.02–5.32	0.44			
Clopidogrel*	0.96	0.59–1.58	0.90			
Prasugrel*	1.43	0.86–2.36	0.19			
Ticlopidine*	0.22	0.03–1.73	0.20			
Statin*	1.55	0.86–2.78	0.16			
Follow-up duration	0.995	0.986–1.004	0.31			
ACS	0.89	0.53–1.50	0.70			
Pre-dilatation balloon maximum diameter (1 mm increase)	1.11	0.75–1.66	0.60			
Pre-dilatation balloon maximum inflation pressure (1 atm increase)	1.04	0.97–1.12	0.26			
Maximum stent diameter (1 mm increase)	1.24	0.69–2.21	0.48			
Minimum stent diameter (1 mm increase)	0.90	0.51–1.60	0.72			
Stent implantation pressure (1 atm increase)	0.97	0.91–1.04	0.43			
Post-dilatation balloon maximum diameter (1 mm increase)	0.51	0.32–0.83	0.006	0.51	0.31–0.86	0.011
Post-dilatation balloon maximum inflation pressure (1 mm increase)	0.93	0.87–0.990	0.048	0.93	0.86–1.001	0.052
At the time of PCI						
Total cholesterol (1 mg/dL increase)	0.995	0.989–1.002	0.18			
Triglyceride (1 mg/dL increase)	1.000	0.998–1.003	0.68			
HDL-cholesterol (1 mg/dL increase)	0.995	0.976–1.014	0.60			
LDL-cholesterol (1 mg/dL increase)	0.994	0/987-1.002	0.15			
At the time of CAS evaluation						
Total cholesterol (1 mg/dL increase)	0.997	0.990–1.005	0.47			
Triglyceride (1 mg/dL increase)	1.001	0.999–1.003	0.50			
HDL-cholesterol (1 mg/dL increase)	0.992	0.975–1.010	0.38			
LDL-cholesterol (1 mg/dL increase)	0.997	0.987–1.007	0.56			

* Medication at the time of coronary angioscopic evaluation.* ACS * acute coronary syndrome,* BMI *body mass index,* CAS*  coronary angioscopy, * DM*   diabetes mellitus,* HDL* high-density lipoprotein,* LDL* low-density lipoprotein,* PCI * percutaneous coronary intervention

**Table 6:** Logistic regression analysis for minimum NIC of Grade 0 (Uncoverage) in patients with DM

	Univariate			Multivariate		
	OR	95% CI	p value	OR	95% CI	p value
Male	1.09	0.39–3.30	1.00			
Age (1 year increase)	1.008	098-1.04	0.65			
BMI	1.04	0.96–1.12	0.35			
Hypertension	1.12	0.37–3.38	1.00			
Dyslipidemia	2.01	0.82–5.14	0.14			
Aspirin*	–	–	–			
Clopidogrel*	1.01	0.51–2.02	1.00			
Prasugrel*	1.15	0.58–2.32	0.72			
Ticlopidine*	0.42	0.048–3.71	0.38			
Statin*	2.11	0.94–4.72	0.088	2.44	1.01–5.87	0.047
Insulin, n (%)	0.82	0.32–2.11	0.82			
Biguanide, n (%)	1.43	0.68–3.03	0.44			
DPP-4 inhibitor, n (%)	1.36	0.68–2.74	0.48			
SGLT-2 inhibitor, n (%)	0.53	0.058-4.90	0.50			
Thiazolidine, n (%)	0.82	0.27–2.44	0.79			
Sulfonylurea, n (%)	2.62	1.23–5.57	0.016	3.87	1.66–9.01	0.002
Glinide, n (%)	0.93	0.23–3.75	0.61			
α-glucosidase inhibitor, n (%)	1.78	0.79–4.04	0.19			
Follow-up duration (1 day increase)	0.995	0.984–1.007	0.44			
ACS	1.13	0.53–2.37	0.85			
Pre-dilatation balloon maximum diameter (1 mm increase)	1.09	0.63–1.90	0.76			
Pre-dilatation balloon maximum inflation pressure (1 atm increase)	1.06	0.96–1.16	0.28			
Maximum stent diameter (1 mm increase)	1.49	0.69–3.20	0.31			
Minimum stent diameter (1 mm increase)	1.13	0.50–2.53	0.77			
Stent implantation pressure (1 atm increase)	0.91	0.81–1.02	0.097	0.87	0.76–0.99	0.040
Post-dilatation balloon maximum diameter (1 mm increase)	0.86	0.43–1.73	0.67			
Post-dilatation balloon maximum inflation pressure (1 mm increase)	0.94	0.84–1.05	0.26			
At the time of PCI						
HbA1c (1% increase)	1.07	0.81–1.42	0.65			
FBS (1 mg/dL increase)	1.001	0.996–1.007	0.67			
CRP (1 mg/dL increase)	1.16	0.84–1.61	0.36			
Total cholesterol (1 mg/dL increase)	0.996	0.987–1.005	0.35			
Triglyceride (1 mg/dL increase)	1.000	0.997–1.003	0.85			
HDL-cholesterol (1 mg/dL increase)	1.022	0.994–1.050	0.12			
LDL-cholesterol (1 mg/dL increase)	0.989	0.978-1.000	0.056	0.984	0.972–0.997	0.018
At the time of CAS evaluation						
HbA1c (1% increase)	1.23	0.82–1.86	0.32			
FBS (1 mg/dL increase)	0.999	0.992–1.005	0.70			
CRP (1 mg/dL increase)	0.53	0.18–1.55	0.53			
Total cholesterol (1 mg/dL increase)	0.999	0.989–1.009	0.81			
Triglyceride (1 mg/dL increase)	1.002	0.999–1.005	0.13			
HDL-cholesterol (1 mg/dL increase)	1.001	0.982–1.021	0.91			
LDL-cholesterol (1 mg/dL increase)	0.990	0.977–1.004	0.17			

* Medication at the time of CAS evaluation.* ACS* acute coronary syndrome,* BMI * body mass index,* CRP* C-reactive protein,* DM* diabetes mellitus,* FBS * fasting blood sugar, HbA_1c_ = Hemoglobin A_1c_,* HDL* high-density lipoprotein,* LDL * low-density lipoprotein

## Discussion

Our findings revealed that (1) the minimum NIC grade was lower in the patients with DM compared to the non-DM patients at 3–5 months after DES implantation; (2) the dominant NIC grade, maximum NIC grade, yellow color grade, and the incidence of thrombus adhesion were similar between the DM and non-DM groups; (3) DM was an independent factor for predicting the minimum NIC of grade 0, which demonstrates uncoverage; (4) in the DM group, the use of sulfonylurea was an independent predictor of the minimum NIC of grade 0 even after the adjustment for confounding factors. To the best of our knowledge, this is the first report describing the relationship between early-phase arterial healing after DES implantation and DM.

Immediately after stent implantation in coronary arteries, bare stent struts are in direct contact with the vessel wall, and the process of arterial healing begins as follows [16, 17]: (1) the first step in arterial healing is the formation of a local thrombus. At the injury site, platelets, fibrin, and red blood cells accumulate and a local thrombus is formed; (2) then, inflammatory cells such as macrophages infiltrate the site; (3) inflammatory cells secrete various growth factors such as platelet-derived growth factor, and smooth muscle cells (SMCs) migrate into the site and begin to proliferate; (4) at 2 weeks after stenting, in addition to the proliferation of SMCs, the extracellular matrix is formed. Neointima formation, that is, neointimal coverage is completed in 12 weeks. The neointima is lined by one layer of endothelial cells which served as an antithrombotic barrier. However, delayed arterial healing sometimes occurs after DES implantation due to the component of DES instead of preventing SMC proliferation, which can lead to in-stent restenosis [18]. A pathological study suggested that widely uncovered struts are a risk factor for stent thrombosis [19], and optical coherence tomography (OCT) studies reported that uncoverage was one of the mechanisms of stent thrombosis [20–22]. There have been several articles which mentioned the neointimal coverage in relation to DM evaluated by OCT. Briguori C et al. elucidated that baseline on-clopidogrel platelet reactivity and complex lesions were independent predictors of uncovered strut rate at 3 months [23]. Kubo T et al. compared the OCT findings between 1st generation sirolimus-eluting stent and 1st generation paclitaxel-eluting stent, and they demonstrated that 1st generation sirolimus-eluting stent showed stronger prohibition of neointimal hyperplasia compared with 1st generation paclitaxel-eluting stent in DM patients as well as in non-DM patients [24]. Kuroda et al. reported that large glucose fluctuations were an independent risk factor for impaired uniform vessel healing after second-generation DES [25]. However, these articles did not compare the early-phase arterial healing between DM and non-DM patients. In the present study, the CAS evaluation demonstrated that the rate of the minimum NIC of grade 0 was significantly higher in the DM group compared to the non-DM group at 3–5 months after DES implantation, which suggests that arterial healing is more delayed in patients with DM compared to those without it.

There are several reports regarding the relationship between the findings of intravascular imaging devices and DM. Kurihara et al. used angioscopy and observed that compared to non-diabetic patients, in pre-diabetic and diabetic patients the number of yellow plaques was greater and the intensity of yellow was greater [26]. They also reported that the number of yellow plaques and the maximum yellow color grade were significantly greater in patients with diabetic retinopathy than in those without it [27]. However, in the present study the yellow color grade was similar between the DM and non-DM groups. Kurihara et al. assessed the CAS findings of the native coronary arteries, whereas we evaluated them 3–5 months after DES implantation. Even with the observation in the relatively early phase after the DES implantation, the difference in the timing of the CAS observations would contribute to the difference in the yellow color grade outcome.

Our present analyses revealed that the post-dilatation balloon size and post-dilatation balloon inflation pressure were the negative predictors of the minimum NIC of grade 0. A study of peripheral arteries showed that the oversized stents caused more neointimal proliferation, which was due to the greater injury to the vessel wall [31]. In addition, malapposition was related to the subsequent incomplete NIC [27]. Adequate strut embedment may cause better neointimal coverage [27, 32, 33]. Since a smaller balloon size and lower inflation pressure would result in less injury to the vessel wall, the difficulty of achieving complete apposition to the vessel wall and inadequate strut embedment, the post-dilatation balloon size and post-dilatation balloon inflation pressure were negatively associated with uncoverage in this study.

Although a previous article revealed that the negative prognostic effect of DM following contemporary PCI was heightened in the presence of insulin treatment [34], insulin therapy did not impact on the early-phase arterial healing in the current study. Instead, we observed that the use of sulfonylurea was an independent predictor of the minimum NIC of grade 0 in the DM patients. It is apparent that aggressive glucose-lowering therapy increased the mortality of DM patients [35], and it has been reported that the use of sulfonylurea itself increased the risk of adverse cardiovascular events [36, 37]. Although the mechanisms underlying the relationship between the use of sulfonylurea and delayed arterial healing after DES implantation in the early phase are not yet understood, it appears that the delayed healing caused by the usage of sulfonylurea may contribute to patients’ poor clinical outcomes. Our present findings also revealed that the glucose control parameters such as the HbA_1c_ had no association with the NIC, and the aggressive glucose control did not impact on the early-phase arterial healing after DES implantation. Although the precise mechanism remains to be undetermined, sulfonylurea treatment should be avoided to prescribe in patients with DM.

There have been several reports which mentioned the relationship between thrombogenicity and DM. Nusca et al. reported that glyco-metabolic state significantly correlated with high platelet reactivity in well-controlled type 2 DM patients on clopidogrel therapy and HbA1c identified patients at higher thrombotic risk but the highest diagnostic accuracy was achieved by combining glycemic variability and HbA1c [38]. Lee et al. mentioned that impaired glucose metabolism was associated with increased thrombin generation potential in patients undergoing PCI [39]. However, the incidence of thrombus adhesion was similar between DM and non-DM patients in the current study. This would be because angioscopic thrombus does not directly mean the thrombogenicity. In other words, angioscopic thrombus adhesion is a benchmark of the completeness of arterial healing because it does not occur where satisfactory arterial healing is achieved [40, 41].

Recent guidelines note that the patient’s bleeding risk and the thrombotic risk should be considered when selecting the duration of DAPT [42–44]. The DAPT score is a landmark of the duration of DAPT performed 1 year after stent implantation, and the presence of DM is one of the factors that encourages the longer DAPT [42]. The PRESICE DAPT score, which evaluates the duration of DAPT at the time of stent implantation, does not include DM as a factor [43]. In the PARIS scoring system, which predicts the risk of thrombotic and bleeding events after discharge based on only the patient’s background, DM is one of the factors that increases the thrombotic risk [44]. A recent European Society of Cardiology guideline also suggests that diffuse lesions in an individual with DM is a risk for stent thrombosis [3]. A diabetic sub-analysis from the PEGASUS-TIMI 54 scribes this point concluding that prolonged DAPT regimens is beneficial in patients with DM [45]. Furthermore, the DAPT score is utilized for prolonged DAPT regimens after one year [42]. Numerous recent trials also have investigated the optimal therapy time for DAPT in non-exclusive DM population below one year in which the results leaves room for debate [46–48]. The patients with DM were low in these trials and therefore these results should not be applied to diabetic patients. In addition, although some clinical trials revealed that the clinical outcomes with short DAPT were non-inferior to those with long DAPT in DM patients, the duration of short DAPT was around 6 months [49, 50]. In the present study, the CAS evaluations demonstrated that the rate of the minimum NIC of grade 0 was significantly higher in the DM group than in the non-DM group 3–5 months after DES implantation, which is consistent with the concept that DM is a factor that increases the thrombotic risk even in the early phase. Clinicians should therefore pay attention to the possibility of switching from DAPT to SAPT in the early phase for patients with DM, and the recent ultra-short DAPT strategy might not be easily applied to DM patients.

## Limitations

This study has several limitations. First, it was a non-randomized, retrospective, observational study; however, the multi-center aspect of the study made the sample size relatively large compared to those of previous studies. Second, an angioscopically observed thrombus does not directly indicate the risk of stent thrombosis. Third, although underlying plaque morphology is associated with vessel healing with neointimal formation, we did not evaluate the baseline lesion morphology by fixed intravascular imaging devices. Forth, since there was a possibility of some differences between 3 months and 5 months after stenting in regard to the NIC, more strict selection of the cases regarding the timing of CAS evaluation would be preferable. However, since the sample size was limited in this retrospective analysis, we cannot help including patients with 3–5 months follow-up. In addition, the follow-up duration was not independently associated with minimum NIC of grade 0 as shown in Table 5. Fifth, the CAS devices were not fixed between these facilities, because this was a retrospective study. Sixth, we included the various type of DES, although it would have a great impact on the results. However, since this was a retrospective study and the sample size was limited, we could not help including the various type of stent, and the type of DES was similar between DM and non-DM groups as shown in Table 4. Seventh, the follow-up time in the DM group was shorter than in the non-DM group and it could affect in the endpoints results. However, in terms of the minimum NIC of Grade 0, follow-up duration did not impact on the result as shown in Table 5. Finally, on some occasions the CAS could not completely evaluate the entire stented segment because of the limitations of the CAS visual field, especially in angulated or tortuous lesions.

## Conclusions

The minimum NIC grade was lower in patients with the DM than in those without DM at 3–5 months after DES implantation, and DM was an independent predictor of the minimum NIC of grade 0, which demonstrates uncoverage, suggesting that the recent ultra-short DAPT strategy might not be easily applied to DM patients.

## Data Availability

The datasets used and analysed during the current study are available from the corresponding author on reasonable request.

## Abbreviations

CAS: Coronary angioscopy
DAPT: Dual-antiplatelet therapy
DES: Drug-eluting stent
DM: Diabetes mellitus
NIC: Neointimal coverage
OCT: Optical coherence tomography
PCI: Percutaneous coronary intervention
SAPT: Single-antiplatelet therapy
